# Seeing the human behind the research: Strengthening emerging African disability researchers

**DOI:** 10.4102/ajod.v13i0.1494

**Published:** 2024-08-29

**Authors:** Michelle Botha, Chioma Ohajunwa

**Affiliations:** 1 Division of Disability and Rehabilitation Studies, Department of Global Health, Faculty of Medicine and Health Sciences, Stellenbosch University, Stellenbosch, South Africa; 2 Africa Centre for HIV and AIDS Management, Faculty of Economic and Management Sciences, Stellenbosch University, Stellenbosch, South Africa

**Keywords:** disability studies, higher education, social justice, conscientisation, African ethic

## Abstract

**Background:**

A pre-conference workshop that investigated the experiences and needs of PhD candidates and early career researchers in disability studies in Africa was held as part of the proceedings of the African Network for Evidence to Action in Disability (AfriNEAD) 7th Conference in November 2023.

**Objectives:**

To determine how the existing structures in AfriINEAD can be leveraged to support emerging African disability researchers. This article documents this event and summarises the key findings from the discussions that took place.

**Method:**

The workshop included presentations from leading scholars in health professions education, panel discussions with PhD candidates and early career researchers, and small group discussions on what is needed to support emerging researchers.

**Results:**

Disability studies was positioned by participants as not only an academic exercise but also a deeply personal pursuit, requiring introspection and conscientisation, with which they felt they needed support. There are also specific ethical concerns related to doing research work with persons with disabilities, which need to be prioritised in postgraduate education in disability studies. The needs identified by participants are summarised as: (1) mentorship, (2) networking, and (3) funding.

**Conclusion:**

We suggest that the development of African disability scholars and scholarship requires an African ethical approach, which prioritises humanity, community and reciprocity.

**Contribution:**

African disability studies scholars are well-placed to disrupt ableism in academic, medical and social spheres, as well as hierarchies within academia, which limit development, mutual growth and respect.

## Introduction

In recent decades, there has been increasing recognition that people with disabilities face particular challenges to access, inclusion and participation in social, economic, political and cultural spheres and that, consequently, a disability-specific focus is required in development intervention planning and implementation (Grech 2009). This move towards greater disability recognition is in no small part because of the persistent activism of movements of people with disabilities, both national and transnational (Conejo 2013; Grech 2009). The United Nations Convention on the Rights of Persons with Disabilities (UNCRPD), which sets a global precedent for the inclusion of people with disabilities in all spheres of society, was, itself, born out of activism of people with disabilities (Conejo 2013; Frohmader & Meekosha 2012). The UNCRPD positions inclusion and participation in such spheres as healthcare, education, employment and law, as well as access to public services and resources, information and communication, and spaces, as inalienable human rights of people with disabilities, representing a decisive shift away from a charitable approach to disability, where inclusion relies on the arbitrary goodwill and altruism of individuals without disability (McClain-Nhlapo 2010). Moreover, the well-known disability rights movement slogan, ‘nothing about us without us’, asserts that people with disabilities are not only rights-bearers but also knowledge-bearers and agents with capacity to make autonomous decisions and effect change (Blackmore & Hodgkins 2012; Charlton 1998).

Despite these important shifts in global disability discourse and legislation, increasing attention has been drawn to the fact that development agendas, programmes and evaluations towards disability inclusion are dominated by theories, models and research from the Global North (Grech 2009; Meekosha 2011; Ned 2022). These critical perspectives call for the recognition of multilayered dynamics of epistemic and political power, between individuals with and without disabilities, resourced and under-resourced contexts, and between other intersecting axes of privilege and oppression operating in specific localities, concerning race, ethnicity and gender, among others (Frohmader & Meekosha 2012; Ned 2022). These dynamics are particularly necessary to understand in relation to disability studies as a distinct discipline concerned with tracing, critiquing, and addressing the social oppression of people with disabilities (Grech 2015; Ned 2022).

Disability studies initially emerged out of activist movements in the United Kingdom and United States, and has, at its foundation, a reconceptualisation of disability as, not only an individual and embodied phenomenon requiring specialist medical interventions and care but also a socio-cultural construct requiring collective political action to promote equality, access and inclusion (Oliver 2004; Shakespeare 2014). While scholars in the Global South do tend to recognise the strides made through this development of disability studies, particularly related to the development of a human rights approach to disability, they register concern that its foundational concepts are often uncritically exported to the Global South, where they may not be either relevant or appropriate (Meekosha 2011; Ned 2022). Northern disability theories, models and research, they contend, tend to assume that the experiences, values and needs of people with disabilities are universal, and are, therefore, out of step with what is often at stake for people with disabilities in the Global South, that is, their very survival in contexts of widespread material deprivation (Grech 2015; Meekosha 2011).

It is obvious that in order for states to promote and implement disability-inclusive programmes and practices, contextually relevant research is required (Grech 2009; Schneider & Suich 2021). This must encompass work that determines disability prevalence, type and severity, the circumstances, needs and lived experience of persons with disabilities, their families, caregivers and communities, as well as the implementation, outcomes and efficacy of policies, programmes and practices to promote disability inclusion in various spheres. There is, therefore, a need to develop disability-aware and inclusive research, and, necessarily, disability-aware researchers, across contexts and academic disciplines such as in education, engineering, health sciences, humanities, law, social sciences, technology, and more. However, this is arguably not possible without the development of robust, contextually focused and appropriate, yet regionally and internationally collaborative disability studies, where context-specific and relevant disability conceptualisations and theories are able to be explored and developed (Ned 2022; Ohajunwa & Sefotho 2024).

In Africa, disability studies continue to steadily gain traction as a legitimate discipline in its own right, evidenced by the growth and development of this publication over recent years, as well as the development of dedicated academic programmes at various universities. Scholars have suggested that the development of disability studies in and for Africa, must involve the incorporation of indigenous knowledge on well-being, health, humanity and disability, and recognition of the disabling impact and epistemic injustice of colonial histories (Ned 2022; Ohajunwa, Mji & Kalenga 2022). It must also recognise the ways in which disability complicates and blurs identity boundaries, scrambling categories of power and oppression in ways that require critical intersectional analysis (Botha & Watermeyer 2021; Chiwandire & Vincent 2019). More than the development of concepts, theories and approaches, a robust, decolonised, context-specific, interdisciplinary and, consequently, transformative disability studies in Africa require the nurturing and strengthening of researchers, both with and without disabilities, across the continent.

With this background established, we report here on some ideas, which emerged from a workshop held with African PhD candidates and early career researchers in disability studies entitled: ‘Towards a community of practice: strengthening emerging researchers in disability studies in Africa’. The workshop formed part of the pre-conference activities of the 7th African Network for Evidence to Action in Disability (AfriNEAD) Conference held in Cape Town, South Africa in November–December 2023. African Network for Evidence to Action in Disability is a pan-African network of scholars, practitioners and activists in the field of disability who are focused on translating research evidence into action (both policy and practice) in order to promote disability inclusion across the continent. The network was founded in 2007 and its activities culminated in a conference, which is held every second year. In this article, we (the workshop organisers and facilitators) offer our reflections on the event and present some overarching ideas on what might be needed to strengthen disability studies and research in Africa based on our learning from the event and post-event debriefing. We contextualise this pre-conference event, including a brief description of the workshop rationale and activities. We also describe elements of an African research ethic with which we frame the further discussion of our impressions of the workshop outcomes and recommendations for future work. Before this, we briefly justify the need for a ‘humanising’ approach in disability studies, particularly in apprehending intersecting subject positions of both researchers and research participants in Africa.

## Humanising disability studies

Disability studies has its roots in collective political action against discrimination and is centred on creating just, inclusive, caring and diverse societies (Goodley, Hughes & Davis 2012; Ned 2022). It requires researchers to think critically about the power-knowledge, which coalesces around disability in various spheres, which necessarily involves apprehending the lived experiences of people with disabilities (which are likely to include inequality and exclusion), as well as confronting societal attitudes and responses towards disability. Freire (1972) first developed the concept of ‘conscientisation’, which has increasingly been drawn upon as a beneficial influence on research practice in disability studies. Conscientisation is a process whereby ‘thinking subjects’ recognise themselves as operating in relationship with the world and, thus, understand that both scholarship and professional practice require a reflexive approach to issues of power, privilege and oppression (Kumagai & Lypson 2009). This process, essential for the promotion of social justice, can be encouraged through education, which intentionally prioritises the development of ‘critical consciousness’ as an outcome (Kumagai & Lypson 2009; Van Schalkwyk & Blitz 2024).

This approach holds particular value for medical, health and rehabilitation professionals who find themselves investigating in the disability field, as relationships between people with disabilities and these professionals have been historically laden with unequal power dynamics, which have often been obscured by medical and charitable model disability theorising, where people with disabilities are seldom recognised as autonomous agents and knowledge-bearers (Botha & Watermeyer 2022; Ebrahim et al. 2020; Oliver 2004). In contrast, the development of critical consciousness involves both thinking and a connection to feelings, and leads ultimately to ‘engaged discourse, collaborative problem-solving, and a “rehumanisation” of human relationships’ (Kumagai & Lypson 2009:783). This holds transformative potential, particularly for health and rehabilitation services (Ebrahim et al. 2020). In addition, there is significance for people with disabilities (both those within and outside of formal academia), and those who are parents, caregivers and loved ones of persons with disabilities, to the development of this critical consciousness as this relates to both individual and collective empowerment. As Charlton (1998:192) asserts of the recognition in disability studies of disability as an issue of social oppression: ‘… to name disability as social oppression is not the defeated wailings of victims, but the clarion call of social change’.

There is resonance here too with other movements to address injustice, such as that based upon gendered, racialised and colonial oppression, which have developed critical pedagogies including critical feminism, critical race theory and decolonial studies as a means to promote critical consciousness and effect social change (Grech 2012; Hook 2012; Tremain 2017). Recognising this resonance is particularly important for the development of disability studies in Africa, which must necessarily deal with intersecting forms of oppression. There is much to be said here about a decolonial imperative, not only in terms of disability studies but also the academy more broadly, as scholars have argued (Grech 2012, 2015; Ndlovu-Gatsheni 2014; Ned 2022). African ways of knowing, doing and being must be central to disability studies teaching and scholarship in Africa, as well as the interventions and practices, which flow from this (Ned 2022; Ohajunwa & Sefotho 2024). Grech (2015) asserts that the decolonial process in disability studies, as well as in other spaces, must involve an intentional recognition of epistemologies and practices from the Global South, and redistribution of power and resources. The decolonial process must also be accepted as messy and continuous and must hold at its centre the recognition of histories of decolonial resistance in particular contexts. Moreover, Grech (2015) asserts that ‘debates and alliances’ are vital to the decolonial project and that within transnational collaborations (including between Global South and Global North):

[*W*]e may paradoxically start to challenge the colonial discourse of Othering and difference, to make fusions productive and, most importantly, non-oppressive, without ever losing focus of the project of eradicating neocolonisation as a historical project transcending spatial and temporal boundaries. (p. 18)

In African disability studies, therefore, there are complicated dynamics of power and privilege, compounded, and in some ways scrambled, by disability as a cross-cutting experience (Ohajunwa et al. 2022). It is necessary, therefore, to pay careful attention to the politics of voice within disability studies, the foregrounding of disability standpoint perspectives, the capacitation of scholars with disability, and the dismantling of disabling barriers within the academy (Chiwandire & Vincent 2019; Dolmage 2017).

## Research methods and design

The pre-conference workshop was held on Wednesday, 29 November 2023. This event aimed to create a space for emerging disability researchers in Africa (including PhD candidates, PhD graduates and post-doctoral fellows) to share knowledge and experiences on the challenges and opportunities that they face as disability researchers in this context. The event organisers were interested to explore the following questions through this event:

- What is needed to strengthen disability theorising for Africa?
- What is needed to develop disability research ethics for Africa?
- What is needed to support the translation of disability research evidence into action and practice in Africa?
- How can the PhD and post-PhD experience be made more meaningful for disability scholars, researchers, practitioners and activists in Africa?

These questions and the rationale for the workshop were developed through several long-form discussions between the event facilitators (the authors of this article) and the chairperson of the AfriNEAD, who commissioned the workshop event, and other board members of AfriNEAD. The event was envisioned as a means to explore how AfriNEAD, as a unique network of scholars, practitioners and activists, can play a role in supporting emerging and early career researchers in the field. This might involve leveraging existing structures of the network and/or looking to future projects. Fundamental to this first event, approached as a sort of ‘fact finding’ mission, and central to this team’s strategising on any subsequent activities, is the recognition that disability studies in and for Africa must necessarily be framed by an African research ethic.

### Workshop structure

The event was structured as a dialogue where the approximately 40 online and in-person attendees were encouraged to engage with a keynote presentation, panel discussions, and small group discussions. The Deputy Vice-Chancellor for Transformation, Student Affairs and Social Responsiveness at the University of Cape Town presented the keynote address. She delivered a strong call to action for health professionals and those involved in health and disability-related research to critically question the dynamics of power-knowledge in their disciplines so as to contribute to dismantling oppression of many kinds in Africa, setting the tone for the discussions that followed.

The bulk of the workshop was taken up with two panel discussions: firstly, with current PhD candidates and secondly with early career researchers, including post-doctoral fellows and junior academic staff. These discussions focused on experiences of challenges and opportunities in navigating doctoral study and the post-doctoral landscape in disability research. Panel questions included the following:

- Why did you feel it was important for you to pursue a doctoral degree?
- What two lessons have you learnt from your journey that you feel are important for those currently pursuing or intending to pursue a doctoral degree?
- How equipped did you feel after completing your doctoral degree to take the next steps in your career?
- Are there skills, competencies, and forms of support that you feel are needed to assist people in navigating the early career stage?

The workshop concluded with a time for small group discussions and feedback on the question: What is needed to build capacity for disability researchers in Africa? Workshop attendees included researchers from contexts across Africa, those with and without disabilities, and from various backgrounds including health professions, social sciences and humanities, law and politics, and education, as well as the disability not-for-profit and activist sectors.

It is important to state here that this article, rather than a piece of formal empirical research, is a reflection of the authors’ lessons from the workshop and the themes, which emerged from the discussions held. This represents an effort to preserve these ideas and feed into the development of future interventions to support emerging and early career researchers through the structures of AfriNEAD. The themes presented in the following section are drawn from the notes taken by both facilitators during the workshop, our post-workshop debriefing discussion, as well as notes taken by participants during the workshop.

Before turning to discuss these findings, we first provide some background to the conceptual framing of this workshop event, which was based on the recognition of the need to develop and promote an African research ethic in disability studies. Crucially, this includes the transformative potential of problematising dominant, Western academic hierarchies and power. There is resonance here too with a feminist research ethic, which has problematised dominant, patriarchal constructions of ‘legitimate knowledge’ towards greater emancipatory research and ‘epistemic justice’ (Fricker 2007; Oakley 1998). Similarly, we argue for an understanding and implementation of research that is informed by an African ethical worldview. The ‘Northernness of disability theory’ (Meekosha 2008:670) has influenced research within the African context predominantly. We present this African ethical moral framework below as a response to this dominant imposition of ideologies of research from the Global North, leaning towards a more syncretic approach and epistemic affirmations.

### An African research ethic

In this article, Africa refers to the sub-Saharan geographical area with more than 3000 tribes with different sub-cultures (Abur & Mugumbate 2022), from the Nubian desert to the Cape of Good Hope and from Senegal to Zanzibar (Nabudere 2005; Ramose 2002 cited in Abur & Mugumbate 2022). While African researchers, here, refer to individuals from the African continent, who currently may live within the continent or who may be visiting other contexts, while conducting academic research on issues related to African contexts, there are varied ontology(ies) and epistemology(ies) that exist within the African continent (Masolo 2019: Metz 2020; Ned 2022; Ohajunwa & Sefotho 2024). However, one prevalent theme connecting these varied understandings is the situating of humanity and human connection as central within these discourses. A key concept undergirding this positioning is the African indigenous concept of Ubuntu, which embodies humanity, community, fairness, equity, reciprocity, among others. This concept can be found within almost every African community in different terms, but with similar meanings (Abur & Mugumbate 2022). Within the African indigenous framework, there is a holistic relationship that exists between the human, other humans, and the physical and spiritual environment around them. The concept of Ubuntu carries this relationship within a two-pronged approach to human engagement (Ohajunwa et al. 2022). As regards the first side of the approach, Ubuntu carries a philosophical and moral mandate to altruism and the greater good to benefit all. This includes the manner and process of knowledge generation, validation, and dissemination, how we do research to benefit society, and not deprive society. Ubuntu calls for the realisation of a higher ethical reason and higher impact for conducting research.

The second side of the twin approach held within the concept of Ubuntu is aligned to the practicalities and strategies of expressing this higher altruism discussed above. Not only does Ubuntu advocate an altruism that should influence research implementation but also provides an ethic of care that addresses the practical considerations of research implementation within the African context. This should occur across all research endeavours, and particularly within disability-related research, which is the focus of this article. Here, the researchers, people with disabilities, funders, various institutions, government, communities, and the physical environment where the research is conducted are recognised as part of this complex ecology of knowledge. Therefore, according to Ubuntu, none should be ignored or reduced within the research engagement. In a bid to avoid any expression of maleficence within this ecology, the practical issues of research and the positioning of the human component of this engagement should be properly considered and planned for. This was a strong theme that emanated from the workshop. Emerging and early career researchers require an ethic of care that can sustain rigorous research, as well as a funding and institutional attitude and process that facilitates collaboration by humanising them, seeing their needs, and valuing their voices. In this way, a reciprocal research relationship is built with intentionality across individuals and institutions within the African context, sharing resources and supporting the other, particularly within the field of disability-related research. This is what we refer to as an African research ethic.

Conceptualising research in this way will, hopefully, assist us to establish sites of knowledge and practice that follows this two-pronged approach within Ubuntu, where knowledge is validated and esteemed, but the bearer of knowledge is not perceived as dispensable, but equally valued.

### Ethical considerations

This article followed all ethical standards for research without direct contact with human or animal subjects.

## Results

The overarching theme that emerged from the discussion can be summarised as the need to ‘see the human behind the research’ – hence its use as the title of this article. This phrase holds a useful dual meaning. Firstly, it captures the need for researchers to ‘see the human’ behind and within their own work – that is, to make decisive efforts to dismantle the historical positioning of people with disabilities as mere ‘objects of study’, rather than active agents and partners in knowledge production (Grech 2015; Kahonde 2023). This focus on safeguarding dignity and agency also aligns with the specifics of what it means to enact ethical research in disability studies in Africa, which requires the recognition and practising of the values of mutual respect, reciprocity and mutually reinforcing humanness (Kahonde 2023; Keikelame 2018). Secondly, ‘seeing the human behind the research’ also draws attention to the researcher herself, as not only involved in producing knowledge as a scholarly product, but as a human being undergoing a, at times, challenging process of personal development related to political and social consciousness (Kumagai & Lypson 2009). This section unpacks this dual imperative towards greater ‘humanity’ in disability studies scholarship. We then turn to the suggestions that workshop participants made for how this can be supported in practical terms, and how disability researchers and research can be strengthened in Africa.

### Seeing the human behind the research

For workshop participants, their work in disability studies was not a process devoid of emotion or personal investment. Indeed, participants shared that their discovery, interest in and entrance into the discipline of disability studies had deep personal and subjective origins. Rather than a response to mere scholarly interest, participants described being drawn into disability studies as a response to particular experiences and the emotions these produced. They used phrases such as: ‘I saw…’ and ‘I felt…’ when explaining the background and rationale to the research they went on to pursue.

This appears to be similar for researchers both with and without disabilities, whether with backgrounds as health professionals, parents and caregivers, activists, and more. The profound impact that encountering disability studies as a distinct discipline can have is illustrated in the following quotation, where a scholar with disability describes her first encounter with disability studies work and its impact as she negotiated her own identity with disability:

At the time, although doing courses in Gender Studies and reading a lot of both Western and African Feminists, I had not yet realised that disability was something that could be theorised beyond the mere fact of a body that did not work properly. I started with Sally French’s *Can You See the Rainbow?* (1993). I must have read it at least five or six times over. I was amazed that a complete stranger could write so profoundly about my own experience. I realized that what I had been doing throughout my teens and into my early twenties was not unique to me. It was called ‘passing’ and I was a seasoned pro. (Botha & Watermeyer 2021:8)

Similarly, the workshop participants described being involved in a process not only of scholarly discovery, but also of developing personal and political consciousness, with which they needed support from supervisors, other more senior academic staff, and peers. The sense from these participants is that, when pursuing disability studies at degree or early career stage, there is ‘the work’, referring to the process of developing academic competencies such as literature searching and reviewing, research design and implementation, and academic writing, and then there is ‘the work’ (articulated with more emphasis). This ‘work’ *refers* to the processes of personal reflexivity – grappling with self-concept and identity, the operation of injustice, as well as one’s own complicity in, and role to disrupt, mechanisms of oppression. This is what is encompassed in the concept, ‘conscientisation’, coined by Freire (1972), as described earlier.

In addition, participants described having to grapple with not only power dynamics between researchers and the communities with whom they engage but also the power-knowledge landscape, which continues to be dominated by theories, methods and practices from the Global North, within which researchers must navigate as scholars and researchers in and from Africa (Grech 2015). In this regard, a strong theme that emerged during the workshop discussions pertained to the particularities of being a researcher from Africa and the sense that individuals might carry some measure of internalised oppression influencing their sense of value and potential. Participants (both with and without disabilities) described the sense that they, like the persons with disabilities on whom their research centres, needed to experience ‘liberation’, in the epistemic sense. This was expressed in terms of the need to feel that they had ‘permission to speak’.

Beyond decolonising epistemologies and methodologies, workshop participants also expressed the need to embrace a decolonial approach to academic relationships, drawing on ubuntu ethics. In this, participants assert that there is a need to foster greater mutual respect, problematise rigid academic hierarchies and promote a more ‘developmental’ approach to scholarship, which holds humanity at its centre. Illustrating this, a participant described the benefit to their doctoral research process of working with a supervisor who, to use their words, ‘had ubuntu in him’. This refers to the sense of being seen, respected and appreciated as a ‘whole person’ within the supervision relationship.

Alongside African scholars’ negotiation of a neocolonial power-knowledge landscape in academia and inextricably intertwined with this, is the need for scholars with disability to navigate ableist power-knowledge mechanisms that silence and exclude – what has been termed ‘academic ableism’ (Dolmage 2017). A participant at the workshop referred to this, particularly as it interacts with disability studies, as the ‘elephant in the room’ – suggesting that the discipline might be more comfortable to look outward to injustice than it is to look inward. This is not to say that the perspectives and expertise of people without disabilities (Swartz 2010), or the exploration of intersecting forms of violence, exclusion and oppression (Bell 2012; Erevelles & Minear 2010) should not be welcome; in fact, these are vital to the development of a robust discipline. What does it imply, although, is the need for careful ethical attention in disability studies, both outward and inward facing, to the particular challenges that scholars with disability are likely to face (Chiwandire & Vincent 2019). Here again, the ubuntu ethic holds potential, capable of containing diverse perspectives, positions, approaches, and the debates that might emanate from these within a framework of mutual respect and humanness.

In addition, the community of practice approach for disability studies described in Lawthom and Chataika (2012), which prioritises individual and community growth and development through the sharing of knowledge and experiences within a group of common interest, warrants more exploration in terms of what it might offer to disability studies in Africa and particularly these complexities of power, voice and epistemic justice (Fricker 2007).

### What is needed to strengthen disability studies researchers in Africa?

The given discussion sheds light on the at times challenging process that disability studies researchers (both with and without disabilities) are navigating. This concerns not only the activities of academic development but also a more internal process of consientisation, including apprehending issues of epistemic injustice (Fricker 2007), related to both the communities in which they are operating, and their own positions within the landscape of academic hierarchies. Workshop participants expressed several ways in which they needed to be supported in negotiating this tricky terrain, which in many ways align with the characteristics that define communities of practice (Lawthom & Chataiga 2012). We share these next, before turning to some final recommendations, with a particular focus on the role of AfriNEAD.

#### Mentorship

There was a sense that far greater interpersonal support was needed in the doctoral and early career phases, particularly given the complexity described here. For participants, this included mentorship from their supervisors, opportunities to engage with other academic staff and focused and intentional engagements with their peers. In the spirit of communities of practice, these mentorship relationships and spaces are envisioned as one of reciprocal learning (Keikelame 2018; Lawthom & Chitaiga 2012). This mentorship must focus on supporting researchers through the process of critical consciousness development, which they are likely to undergo in their journey with disability studies scholarship. The sense from participants is that mentorship at several levels would combat anxieties and isolation, which accompany this deeply personal, internal work.

Mentorship is also necessary to provide support with developing the competencies required in academic work, with which some participants felt they were not sufficiently prepared when moving into doctoral study. For instance, a participant described the sense that when starting a doctoral degree, the expectation from supervisors and other academic staff seems to be that ‘you already know what to do’. Given that disability studies attract people from a variety of backgrounds, including disability rights activism, direct service provision in the not-for-profit sector, health and rehabilitation professions, and more, it is necessary to ensure that candidates are prepared, whether to make the transition back into academia after a significant break, or to move from clinical disciplines into the more social sciences-oriented field of disability studies. In addition, the very particular ethical considerations related to doing research with people with disabilities were identified during the workshop as an area requiring development, and with which participants felt they needed guidance. In particular, the need to carefully consider participation of people with disabilities within the research process, as well as how best to translate and disseminate research findings to benefit communities were mentioned.

A third area of concern where participants felt that more intentional mentorship was required was the development of a clear ‘exit plan’ with their supervisors. Similar to the above-mentioned statement, participants expressed that it was simply assumed that they knew how academic hierarchies, advancement and career planning and development work. Instead, this was an area where they felt the need for more information, guidance and support. Again, it was felt that both peer-to-peer and supervision mentorship in these more practical areas of academic development and advancement would assist greatly to combatting anxiety and isolation, as a participant put it, to offer ‘hope’.

It was felt that formalised pre-doctoral support would also aid these processes of scholarly and personal development, but that there are funding implications to this which would need to be negotiated. Pre-doctoral training is increasingly recognised as an important ‘bridging’ stage where doctoral candidates are equipped with information, opportunities to begin to develop requisite skills and competencies, and time to build rapport with a supervisor and network within the university (Chan 2008). Furthermore, research has suggested, a key time to build resilience and to delve into a reflexive process concerning the researcher’s role and positions of power and privilege (Chan 2008; McKenzie, Kent & Valero 2022).

#### Networking

Participants recognised the importance of developing strong networks across disciplines and geographies. It was felt that pan-African networks were important to maintain a sense of the work that is happening in disability studies on the continent, particularly to avoid duplication and encourage collaboration. It was also felt that these networks would assist in the translation and dissemination of research findings into action for communities, connected to the concern raised here. Moreover, it has been suggested that collaboration, particularly between the Global North and Global South, is important in promoting the decolonial project in disability studies – encouraging the greater global recognition of voices, knowledge and approaches emanating from the Global South, and capacitating researchers and institutions through partnership (Grech 2015).

There was a sense in the workshop discussions that disability studies researchers, regardless of context, shared a connection rooted in the belief in the liberatory and transformative goals of the discipline, as a participant expressed, ‘we speak the same language’. However, they were also clear that this connection, and resulting collaboration, must not obscure or undermine context-specific realities and concerns. There was also the sense of a deeply felt responsibility of disability studies researchers to be active within their background disciplines to dismantle oppressive concepts and practices and to advocate for disability inclusion and participation. This difficult work, which might require people to occupy the role of ‘disrupter’, cannot be performed in isolation and would be strengthened through both pan-African and global disability studies and research networks.

#### Funding

Unsurprisingly, a key concern raised during the workshop discussions, and an area where participants felt they needed more support, was funding. Participants referred not only to funding through bursaries, scholarships and research grants but also through being afforded opportunities to earn through teaching, supervising, and other work opportunities within the departments in which they are registered. In these discussions on funding, layered socio-economic vulnerabilities, specific to under-resourced contexts in the Global South, come into view. For instance, a participant described their situation as ‘living from hand to mouth’. There are also specific challenges to entering and remaining in tertiary education for students with disabilities, which include insufficient designated funding mechanisms to support this group (Chiwandire & Vincent 2019).

A further concern regarding funding relates to the agenda setting power that funding agencies hold. This is particularly relevant to partnerships with funders in the Global North, who may be out of touch with context-specific norms, circumstances, and needs (Grech 2009). Aligned with the sentiments of participants already shared, the workshop discussions also called for the decolonisation of the funding landscape, referring in particular to the need for local agendas to lead the way in the development of funding priorities, grant allocations, and research and/or intervention planning and implementation (Grech 2009).

These three needs, namely: (1) mentorship, (2) networking, and (3) funding are interconnected and reliant on each other in various ways. For instance, negotiating the complexities of the funding landscape can be facilitated through mentorship and networking. Similarly, networking and mentoring opportunities can be facilitated through designated funding. Our suggestion, emanating from this workshop event, is that these should be viewed as the necessary pillars of doctoral and early career support to strengthen researchers and research in disability studies in Africa. These support pillars, and activities to develop them, should equally be informed by the overarching principles related to social justice and humanity discussed earlier. African Network for Evidence to Action in Disability has a significant role to play here.

## Discussion

In the interest of a sustainable research engagement that takes note of the unique challenges experienced within disability-related research and researchers within the African context, there must be intentionality within the approaches utilised by individuals and institutions.

African Network for Evidence to Action in Disability, by virtue of its position as a network of institutions, non-governmental organisations (NGOs), and advocates, should continue to create spaces where researchers and advocates can meet with each other and network. The seventh AfriNEAD pre-conference was aimed at this, bringing in academics to talk across disciplines and further develop transdisciplinary discourse related to disability research.

The AfriNEAD has country working groups, with affiliations across universities and civil society organisations across the continent. The country-working groups should be strategically involved in targeted advocacy to enhance collaborative research that benefits and supports both the growth and retainment of early career researchers within our institutions across the continent.

Higher education institutions in Africa must recognise the need to support career pathways for PhD and post-doctoral fellows, by exploring various ways and means to support their sustained contribution to the academic institutions.

A decolonisation of the mind and the research processes, where collaborators from the Global North are often more sought after than Africa-wide collaborators must be addressed. A more developmental approach must be used within institutions to mentor emerging and early career academics, respecting and valuing the unique contributions that they make as the future of academia within the continent.

The government and their agents, who often negotiate with funders, must begin to bring the voices of early career and emerging academics to the negotiating table. This is especially critical when related to disability research, where there has been historic marginalisation and imposition of northern ideologies of the disability experience within research in Africa. This imposition is often spurred on by international funders.

## Conclusion

In this article, we presented the outcomes of a pre-conference workshop held as part of the 7th AfriNEAD conference, focusing on the experiences and needs of PhD candidates and early career researchers in disability studies in Africa. The pre-conference was aimed at engaging with these academics to understand their unique positioning and experiences. These academics face unique challenges linked to a shared history of oppression and ongoing coloniality that impact their experiences as academics, and the future of academia in Africa. The dominant understanding of disability studies itself, as a discipline, emerges from northern thought, and mandate from funders, further subjugate the realities for African disability studies researchers. Participants emphasised the need for support and regulation in terms of showing appropriate sensitivity and conscientisation when it comes to disability research within the continent. The three main areas of challenge highlighted by participants are mentoring, networking and funding. These three areas, although implemented through various systems, reflect a thread of human engagement and support, that is purported by Ubuntu. As advocated by Ubuntu, the challenges of retainment and employment by higher academic institutions, decolonising the research process by focusing on contextually relevant and sustainable frameworks, the provision of mentoring in a respectful manner that values rather than subjugate the other, advocacy and fair negotiations with funders who often influence policy and research directives, can all be done while centring the humanity of the researcher within these multiple processes of disability research engagement.
